# Is an Endorectal Balloon Beneficial for Rectal Sparing after Spacer Implantation in Prostate Cancer Patients Treated with Hypofractionated Intensity-Modulated Proton Beam Therapy? A Dosimetric and Radiobiological Comparison Study

**DOI:** 10.3390/curroncol30010058

**Published:** 2023-01-06

**Authors:** Dalia Ahmad Khalil, Jörg Wulff, Danny Jazmati, Dirk Geismar, Christian Bäumer, Paul-Heinz Kramer, Theresa Steinmeier, Stefanie Schulze Schleithoff, Stephan Tschirdewahn, Boris Hadaschik, Beate Timmermann

**Affiliations:** 1 Department of Particle Therapy, University Hospital Essen, West German Proton Therapy Centre Essen (WPE), West German Cancer Center (WTZ), 45147 Essen, Germany; 2 Faculty of Physics, TU Dortmund University, 44227 Dortmund, Germany; 3 Department of Urology, University Hospital Essen, University of Duisburg-Essen, 45147 Essen, Germany; 4 German Cancer Consortium (DKTK), 45147 Essen, Germany

**Keywords:** endorectal balloon, proton therapy, intensity-modulated therapy, prostate cancer, dose-escalated radiation therapy, hypofractionated radiation therapy

## Abstract

Background: The aim of this study is to examine the dosimetric influence of endorectal balloons (ERB) on rectal sparing in prostate cancer patients with implanted hydrogel rectum spacers treated with dose-escalated or hypofractionated intensity-modulated proton beam therapy (IMPT). Methods: Ten patients with localized prostate cancer included in the ProRegPros study and treated at our center were investigated. All patients underwent placement of hydrogel rectum spacers before planning. Two planning CTs (with and without 120 cm^3^ fluid-filled ERB) were applied for each patient. Dose prescription was set according to the h strategy, with 72 Gray (Gy)/2.4 Gy/5× weekly to prostate + 1 cm of the seminal vesicle, and 60 Gy/2 Gy/5× weekly to prostate + 2 cm of the seminal vesicle. Planning with two laterally opposed IMPT beams was performed in both CTs. Rectal dosimetry values including dose-volume statistics and normal tissue complication probability (NTCP) were compared for both plans (non-ERB plans vs. ERB plans). Results: For ERB plans compared with non-ERB, the reductions were 8.51 ± 5.25 Gy (RBE) (*p* = 0.000) and 15.76 ± 11.11 Gy (*p* = 0.001) for the mean and the median rectal doses, respectively. No significant reductions in rectal volumes were found after high dose levels. The use of ERB resulted in significant reduction in rectal volume after receiving 50 Gy (RBE), 40 Gy (RBE), 30 Gy (RBE), 20 Gy (RBE), and 10 Gy (RBE) with *p* values of 0.034, 0.008, 0.003, 0.001, and 0.001, respectively. No differences between ERB and non-ERB plans for the anterior rectum were observed. ERB reduced posterior rectal volumes in patients who received 30 Gy (RBE), 20 Gy (RBE), or 10 Gy (RBE), with *p* values of 0.019, 0.003, and 0.001, respectively. According to the NTCP models, no significant reductions were observed in mean or median rectal toxicity (late rectal bleeding ≥ 2, necrosis or stenosis, and late rectal toxicity ≥ 3) when using the ERB. Conclusion: ERB reduced rectal volumes exposed to intermediate or low dose levels. However, no significant reduction in rectal volume was observed in patients receiving high or intermediate doses. There was no benefit and also no disadvantage associated with the use of ERB for late rectal toxicity, according to available NTCP models.

## 1. Introduction

Despite advances in radiotherapy (RT) techniques, rectal morbidity related to prostate radiation treatment cannot be entirely avoided and carries implications for quality of life (QOL). Escalation of radiation dosage for prostate cancer patients has evolved over the past decade with the development of modern three-dimensional conformal radiotherapy (3D-CRT) and the more advanced intensity-modulated radiotherapy (IMRT) together with image-guided radiotherapy (IGRT). Several randomized studies have demonstrated that dose escalation offers improved local control and biochemical control rates compared with conventional doses [1,2,3,4,5,6]. However, the relative biological effectiveness (RBE) achieved by escalating the total dose delivered to the prostate by 8–10 Gray (Gy) has been shown to significantly increase the risk of rectal toxicity by about 10% [2,7,8,9]. The results of several trials have been published relating rectal dose-volume characteristics to radiotherapy-induced rectal toxicity [10,11,12,13,14]. Based on these reports, the efforts of radiation oncologists in the past decade have been directed not only towards utilizing modern radiotherapy techniques for patients with prostate cancer, but also to incorporating mechanical tools to increase the separation between prostate and rectum, such as implantation of rectum–prostate spacers and/or the use of endorectal balloons (ERBs).

Significant reduction of intra-fractional prostate motion during radiotherapy achieved by using ERB was shown in a systematic review of 21 articles [15]. The dosimetric effect of ERB in reducing rectal radiation exposure during 3D-CRT, IMRT, or stereotactic body radiation therapy for prostate cancer has been demonstrated in several studies [16,17,18,19,20,21,22,23]. However, there have been few trials in the field of proton therapy that have investigated the role of ERB [24,25,26,27,28].

The pencil beam scanning (PBS) technique is highly sensitive to organ motion [29], therefore ERB has more frequently been used in our institution to stabilize the position and shape of the rectum and hence fix the position of the prostate during treatment. It is unclear whether the benefit of ERB is retained when decreasing the dose exposure in the rectum. Our goal was to explore the dosimetric impact of ERB on rectal dosage and normal tissue complication probability (NTCP) values in prostate cancer patients with implanted rectum spacers who were treated with dose-escalated or hypofractionated IMPT. 

## 2. Materials and Methods

Since August 2015, a prospective single-center register evaluating proton therapy for patients with localized prostate cancer (ProRegPros) has been carried out at the West German Proton Therapy Centre Essen (WPE). Two computed tomography (CT) scans, respectively before and after the insertion of the ERB, were obtained for each of 10 consecutive patients undergoing prostate cancer treatment. All patients had been diagnosed with T1–T4, N0, M0, and PSA ≤ 50 ng/mL. All patients were treated with dose escalated or moderate hypofractionated IMPT with 72 Gy (RBE) in 30 fractions. All the patients had been diagnosed with intermediate- to high-risk prostate symptoms (T1–T4, N0, M0, PSA ≤ 50 ng/mL, Gleason score 7a–9) and had no indication of lymph node irradiation. Patients were in good general health with no life-limiting conditions, and each had a life expectancy of more than five years.

All patients selected for analysis underwent hydrogel rectal spacer insertion and fiducial marker implantation one week before planned CT application. 

Written informed consent was obtained from all patients for their inclusion in the register. The register was approved by the ethical committee of the University of Duisburg Essen.

### 2.1. CT-MRI Simulation

All patients drank 350 mL water on an empty bladder 30 min prior to simulation. Patients were immobilized in a supine position using a thermoplastic pelvic cast. The first planning CT was acquired in 1 mm slices for each patient. Then, the thermoplastic pelvic cast was removed and the ERB catheter was inserted with the patient in a knees-raised position, then the catheter was filled with 120 cm^3^ of fluid. The patient was positioned and immobilized again using the laser alignment and immobilization mask markings placed during the first CT, and the second CT was acquired in 1 mm slices. A T1/T2-weighted MRI scan was performed for each patient. 

### 2.2. Target Volumes and OARs Delineation

Within our in-house standard framework, taking into account national and international recommendations and guidelines, we determined target volume and dose. Planning and contouring for each patient were performed using the same methods. For each patient in every CT, the prostate, seminal vesicles, clinical target volumes (CTV,) and organs at risk (OARs) were contoured using a combination of CT and magnetic resonance imaging (MRI) for accurate prostate delineation. Two CTVs were defined; low risk CTV1 (prostate + 5 mm peri-prostatic tissue + 2 cm of the seminal vesicles), and high risk CTV2 (prostate + 1 cm of the seminal vesicles). Margins of 5 mm in every direction were added to the CTV to create the corresponding planning target volumes (PTVs), except at the seminal vesicle region where a 7 mm margin was applied [29]. Dose prescription was 60 Gy (RBE) in 2 Gy to PTV1 and 72 Gy (RBE) in 2.4 Gy to PTV2, in 30 fractions using simultaneous integrated boost (SIB). The rectum was contoured as a solid organ extending from just above the anal verge to the sigmoid flexure. Extra contours were generated for the anterior and posterior rectum. 

### 2.3. SIB-IMPT Planning Process

Dose calculation and optimization of IMPT plans were performed using a pencil beam algorithm with the RayStation treatment planning system version 6 (RaySearch Laboratories, Stockholm, Sweden). For all patients, fixed geometry plans were generated in both CTs using two laterally opposed IMPT beams with the same optimization goals. A margin of 3.5% proton beam range + 2 mm was included in the PTV in the beam direction to account for field-specific range uncertainty. For greater consistency, all contours were generated by the same senior radiation oncologist who also created all the treatment plans. For all dose concepts, a generic relative biological effectiveness (RBE) factor of 1.1 (relative to that of Co-60) was assumed.

### 2.4. DVH Analysis and Rectal NTCP Calculation

The dose-volume histogram (DVH) of the rectum was assessed and the following parameters were calculated:

- For the whole rectum: RV (rectal volume in cc), Dmax, Dmean, Dmedian, and RVxGy = percentage of rectal volume received X dose in Gy (RV72Gy, RV70Gy, RV65Gy, RV60Gy, RV55Gy, RV50Gy, RV40Gy, RV30Gy, RV20Gy, and RV10Gy).
- For the anterior rectum: Dmax and Ant-RVxGy = percentage of anterior rectal volume received x dose in Gy.
- For the posterior rectum: Dmax and Post-RVxGy = percentage of posterior rectal volume received x dose in Gy.

NTCPs are able to predict the toxicity of radiation therapy to organs at risk. These biological models can be used to predict the risk of various complications.

For the rectal NTCP calculation, the following biological models available in RayStation were employed:

- Layman Kutcher Burman (LKB) model for late rectal bleeding ≥ 2 with D50 = 81.8 Gy, γ = 3, *m* = 0.22, *n* = 0.29, and α/β = 3 [30].
- Poisson-LQ model for necrosis or stenosis with D50 = 80 Gy, γ = 2.2, *S* = 1, and α/β = 3 [27].
- LKB model for late effects grade ≥ 3 with D50 = 80 Gy, *m* = 0.15, *n* = 0.06, and α/β = 3.9 [31].

We compared the rectal DVH parameters and rectal NTCP values of the non-rectal balloon plans (non-ERB group) with those of the rectal balloon plans (ERB group). The differences in DVH and NTCP indices were calculated (Δ = mean value of non-ERB plans − mean value of ERB plans). Statistical analysis was conducted using the IBM SPSS Statistics program V22. The Mann–Whitney U test was applied to compare means between the non-ERB and ERB plans. 

## 3. Results

### 3.1. DVH Analysis

**Figure 1:**
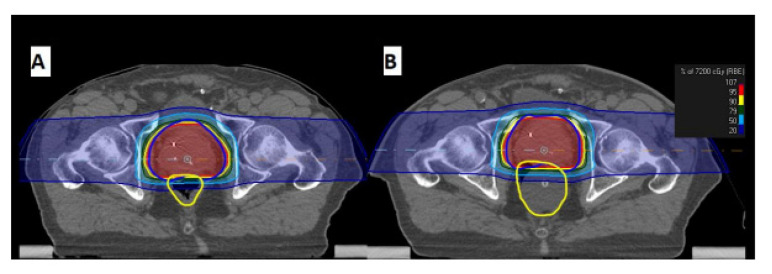
Example of IMPT dose distribution for cT2N0M0 Prostate cancer patient. (**A**) non-ERB plan. (**B**) ERB plan.

The 120 cm^3^ fluid-filled ERBs significantly increased rectal volume in ERB patients compared to non-ERB patients. Analysis of the DVH of the whole rectum confirmed that the ERB plans could attain lower values of Dmax, D1, Dmean, and Dmedian in comparison with non-ERB plans. However, the differences in Dmax and D1 were not statistically significant. There was a minimal statistically insignificant reduction in RV72Gy in favor of non-ERB plans compared with ERB. Otherwise, the ERB plans were able to lower the rectal volumes exposed to different radiation doses compared with the non-ERB plans, with an insignificant reduction in RV70Gy, RV65Gy, RV60Gy, and RV55Gy and a significant reduction in RV50Gy, RV40Gy, RV30Gy, RV20Gy, and RV10Gy (Table 1, Figure 1).

In the results of the analysis carried out for the anterior rectum, we found that ERB reduced the values of Dmax, D1, RV72Gy, RV70Gy, RV65Gy, RV60Gy, RV55G, RV50G, RV40G, RV30Gy, RV20Gy, and RV10Gy, but no statistically significant differences were attained (Table 2).

**Figure 2:**
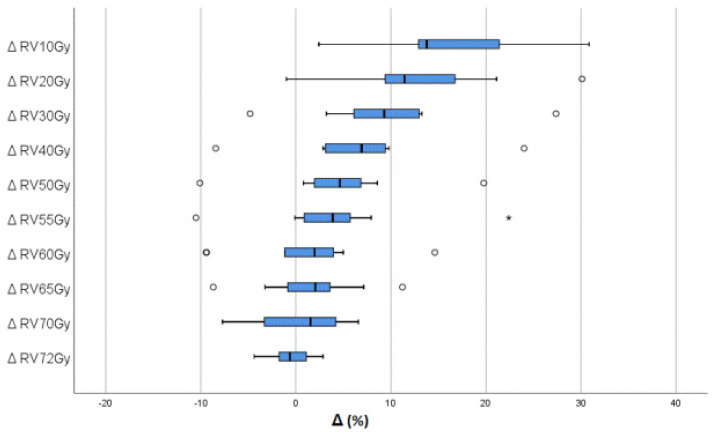
Box plot illustrates the difference (Δ) in percentage of rectal volume received x dose between the non-ERB plans and ERB plans (mean value non-ERB plans- mean value ERB plans).

For the posterior rectum, the Dmax and D1 were reduced in ERB plans in comparison with non-ERB plans, without statistical significance. There were no statistically significant differences between the two groups in terms of RV72Gy, RV70G, RV65Gy, RV60Gy, RV55Gy, or RV40Gy (Figure 2). Statistically significant differences were found between the two groups for rectal volumes after receiving 30 Gy, 20 Gy, and 10 Gy (Table 3).

### 3.2. NTCP Results

**Figure 3:**
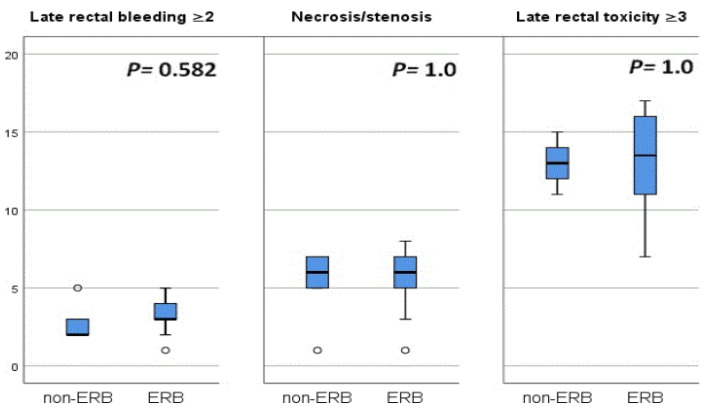
Blox plot comparing median and full range of variation of the rectal NTCP rates for late rectal bleeding ≥2, necrosis/stenosis, and late toxicity ≥3 for non ERB plans vs. ERB plans.

No statistically significant differences between the two study groups were determined for the risk of NTCP with late rectal toxicities. Comparisons of NTCP results for late rectal bleeding ≥ 2, necrosis or stenosis, and late rectal toxicity ≥ 3 are presented in Table 4 and Figure 3.

## 4. Discussion

Few trials have been conducted into the use of proton therapy for prostate patients in order to investigate the effectiveness of ERB utilization to achieve rectal sparing [24], reduction of the interfraction prostate motion [26], or removal of rectal gas [25]. Our aim was to investigate whether insertion of 120 cm^3^ fluid-filled ERB could spare rectal space and hence reduce rectal NTCPs in patients who had undergone prior placement of hydrogel rectal spacers and received treatment with dose-escalated or hypofractionated IMPT to the prostate and the seminal vesicle.

In this study, ERB increased the rectal volume by 137.35 ± 32.58 cm^3^. The reduction in mean radiation dose received by the whole rectum in the ERB plans compared to non-ERB plans was 8.51± 5.25 Gy (RBE) (*p* = 0.000), and for Dmedian the reduction was 15.76 ± 11.11 Gy (RBE) (*p* = 0.001). Regarding the maximum dose delivered to the rectum, we recorded a 0.64 Gy (RBE) difference in Dmax, a 0.11 Gy (RBE) difference in D1 of the rectum, and a 0.21 Gy difference in D1 of the anterior rectum in favor of the ERB plans, but with no statistical significance. We found that ERB could reduce Dmax in the posterior rectum, but with no statistical significance. Furthermore, D1 was reduced in ERB plans by 11.11 ± 13.93 Gy (RBE) with a marginal statistical significance (*p* = 0.059). Our results are similar to those reported by Elsayed et al., who applied 3D-CRT with 59.4 Gy (RBE) + 10 Gy (RBE) high dose-rate (HDR) brachytherapy to 12 patients. The authors found that for tele-therapy applied with a PTV including prostate + 9 mm safety margins, the application of a 60 cm^3^ air-filled ERB led to a decrease in Dmax of the anterior rectal wall and the rectum as a complete organ, but with no statistical significance. However, owing to the dose distribution obtained from the 3D-CRT, the authors demonstrated a reduction in the Dmax of the posterior rectal wall of 18.6 Gy (RBE) (47.1 Gy for non-ERB vs. 28.5 Gy for ERB), which was found to be significant (*p* = 0.01) [32].

Regarding rectal volumes receiving different dose levels, we found no statistically significant differences of rectal volumes at high or intermediate dose levels. Furthermore, through separate analysis of the anterior rectum, we found that the ERB plans led to no significant differences in comparison with non-ERB plans in any of the DVH parameters examined. In the case of intermediate and low dosage levels, the differences in rectal volume between non-ERB and ERB plans were found to be 4.58, 6.82, 9.57, 12.87, and 15.78% for RV50Gy (RBE), RV40Gy (RBE), RV30Gy (RBE), RV20Gy (RBE), and RV10Gy (RBE), respectively, which were statistically significant. Further analysis of the posterior rectum confirmed that the ERB reduced Post-RV30Gy (RBE) by 8.89 ± 9.92% (*p* = 0.019), Post-RV20Gy by 15.76 ± 12.94% (*p* = 0.003), and the Post-RV10Gy by 25.66 ± 14.21% (*p* = 0.001).

Our results are in agreement with those reported by Hille et al., who used 3D-CRT and applied 72 Gy with conventional fractionation. The authors found that after inclusion of the prostate, the entire, and the proximal seminal vesicles as CTV, a 60 cm^3^ air-filled ERB led to a significant decrease of the rectal wall receiving 40 Gy and 50 Gy, while no significant decrease of the rectal wall receiving 60 Gy, 65 Gy, or 70 Gy could be found [33].

Other trials applying 3D-CRT demonstrated that insertion of ERB could lower rectal volumes exposed to high doses. In an early study in 2002, Wachter et al. used 3D-CRT with 66 Gy for prostate cancer, and tested the role of a 40 cm^3^ air-filled ERB on the rectal dose. The authors found that for PTV prostate-only plans, the proportion of the rectum volume receiving doses larger than 90% could be reduced from 24% without ERB to 20% with ERB. However, for PTV prostate + seminal vesicle plans, the volume increased from 41% without ERB to 48% with ERB, due to posterior displacement of the seminal vesicle resulting from application of the ERB [34]. Van Lin et al. conducted a study testing 40, 80, and 100 cm^3^ air-filled ERB vs. non-ERB plans, using three-dimensional conformal radiation therapy (DCRT) and IMRT delivered to two different PTVs with and without seminal vesicle involvement. They found that in cases of 3D-CRT the application of an ERB resulted in a statistically significant reduction of the mean rectal wall dose, which was the case for rectal wall volume irradiated to a dose level of 70 Gy or more and for that irradiated to a dose level of 50 Gy or more. However, in case of IMRT, the authors reported no statistically significant reduction in the rectal wall dose parameters for any of the ERBs [16]. In contrast the results obtained by Van Lin et al., Patel et al. conducted a planning study to detect the beneficial effect of 60 cm^3^ air-filled ERB on rectal dosimetry. They generated radiotherapy plans for five patients, delivering 76 Gy either with 3DCRT or IMRT to target volumes with and without inclusion of the seminal vesicle, and proved that inflation of the ERB in all cases and even in the context of IMRT resulted in significant decreases in the absolute volume of rectal wall receiving greater than 60, 65, or 70 Gy [23].

Vargas et al. published the only trial to have investigated the role of ERB in rectal sparing for patients treated with proton therapy. They analyzed 20 proton plans for 15 patients who received doses of 78–82 Gy, and found that ERB decreased the volume of the rectum radiated by doses from 10 to 65 Gy (*p* ≤ 0.05), while no benefit was observed for doses ≥ 70 Gy [24]. No hydrogel prostate rectum spacers were used in their trial.

Based on NTCP calculations, we found that the probability of late rectal toxicity was not reduced by the application of ERB. The mean NTCP for late rectal bleeding ≥ grade 2 was 2.6 ± 0.97% for non-ERB plans vs. 3.1± 1.1% for ERB (*p* = 0.15). For necrosis or stenosis it was 5.5 ± 1.78% for non-ERB vs. 5.6 ± 2.22% for ERB (*p* = 0.72); for late rectal toxicity ≥ 3 it was 13.1 ± 1.37% for non-ERB vs. 13.3 ± 3.02% for ERB (*p* = 0.593). Our results are similar to those reported by Van Lin et al., who used the LKB model with Emami parameters (n = 0.12, m = 0.15, and D50 = 80 Gy) for calculation of late rectal NTCP. In their trial, no statistically significant reduction in NTCP could be demonstrated for the combination of IMRT with ERBs (40, 80, and 100 cm^3^ air-filled). However, according to their analysis, ERB could improve the results of 3D-CRT plans, with a statistically significant reduction in rectal NTCP for 100 cm^3^ air-filled ERB compared to non-ERB (15% vs. 24%, respectively, *p* < 0.0001) [16].

It has been proven that the exposure of rectal volume to intermediate and high radiation doses is associated with developing late rectal toxicities. Storey et al. reported a significant correlation between the percentage of the rectum irradiated to 70 Gy or greater and the likelihood of developing late rectal complications in patients treated with up to 78 Gy [35]. Kupelian et al. tested a short-course IMRT (70 Gy with 2.5 Gy per fraction) and demonstrated that only the volume of rectum receiving 70 Gy (with a cutoff of 15 cc) was a significant predictor of rectal bleeding [11]. Huang et al. also observed a significant effect on volume at rectal doses of 60, 70, 75.6, and 78 Gy and concluded that the risk of developing rectal complications increased exponentially as larger volumes were irradiated [36]. Zapatero et al. reported that rectal Dmean and the percentage of the rectum receiving >60 Gy were correlated with grade 2 rectal bleeding or worse [37]. Meanwhile, other investigators have demonstrated the likelihood of rectal toxicity for rectal volumes receiving an intermediate dose. Tucker and colleagues found that the incidence of grade 2 or worse late rectal bleeding increased within 2 years when ≥80% of the rectal wall was exposed to doses > 32 Gy [38]. Jackson et al. reported that rectal bleeding was significantly correlated with volumes exposed to 46 Gy in prostate cancer patients who received 70.2 or 75.6 Gy [39].

The strength of the current study is limited by the small number of patients involved. Nevertheless, since the data include internal controls, the dataset is particularly homogeneous and thus highly relevant.

## 5. Conclusions

Our study suggests that ERB could reduce rectal volumes exposed to intermediate or low doses of radiation treatment in prostate cancer patients with implanted rectum spacers during their treatment with hypofractionated or dose-escalated IMPT. We could not find any benefit associated with ERB in terms of reducing rectal volumes receiving high to intermediate dose levels. Supported by previous trials, these results can explain the lack of benefit obtained from ERB in reducing NTCP values for late rectal toxicity in those patients. We conclude that the application of ERB adds little benefit for patients treated with IMPT, due to high capability of this technique to conform the dose to the target, which in turn reduces the volume of the rectum exposed to high doses. Furthermore, reduction of the rectum volume receiving a high dose can be achieved using spacer implantation. However, the potential effect of ERB in reducing volumetric changes in the rectum cannot be neglected, and nor can variabilities in rectal positioning during treatment, especially in patients undergoing proton therapy due to the high sensitivity of PBS dose distribution to inter- and intrafractional motion. This issue is currently under investigation at our center, and results will be reported soon. Therefore, at our center we are currently continuing to use the endorectal balloon to reduce motion.

## Figures and Tables

**Table 1:** DVH analysis for the whole rectum comparing between the non-ERB plans and ERB plans.

	Study Group	Mean	SD	Range	Diff (Δ) ^d^		*p* Value
					Mean	SD	
RV ^a^	non-ERB ERB	90.79 228.14	42.34 31.65	42.08–183.51 179.79–267.54	137.35	32.58	0.000
D_max_^b^	non-ERB ERB	73.62 72.98	0.77 0.55	72.6–74.8 72.6–74.4	0.64	0.85	0.103
D_mean_^b^	non-ERB ERB	31.42 22.91	4.98 3.0	24.20–42.29 18.79–27.53	8.51	5.25	0.000
D_median_^b^	non-ERB ERB	26.34 10.57	9.66 5.89	13.06–45.25 4.39–21.52	15.76	11.11	0.001
D1 ^b^	non-ERB ERB	72.49 72.38	0.53 0.16	71.59–73.29 72.21–72.78	0.11	0.52	0.363
RV72Gy ^c^	non-ERB ERB	3.42 3.80	1.73 1.07	0.34–5.60 1.96–5.30	−0.38	2.24	0.734
RV70Gy ^c^	non-ERB ERB	7.90 7.32	3.4 2.03	2.12–13.29 3.89–9.84	0.58	4.42	0.597
RV65Gy ^c^	non-ERB ERB	13.98 12.31	4.3 2.54	5.93–21.28 7.66–15.98	1.66	5.45	0.257
RV60Gy ^c^	non-ERB ERB	18.74 17.64	4.85 5.62	9.63–27.32 11.17–31.51	1.10	6.99	0.345
RV55Gy ^c^	non-ERB ERB	23.37 19.35	6.37 3.15	13.11–37.72 14.85–23.96	4.02	8.15	0.082
RV50Gy ^c^	non-ERB ERB	26.87 22.29	5.59 3.43	16.49–37.51 17.52–27.51	4.58	7.37	0.034
RV40Gy ^c^	non-ERB ERB	34.50 27.68	6.19 3.74	23.45–46.37 22.36–33.83	6.82	8.02	0.008
RV30Gy ^c^	non-ERB ERB	42.67 33.10	6.60 4.4	32.64–54.68 27.05–40.37	9.57	8.23	0.003
RV20Gy^c^	non-ERB ERB	52.33 39.46	7.75 5.09	42.85–65.88 32.58–47.58	12.87	8.75	0.001
RV10Gy ^c^	non-ERB ERB	64.57 48.77	8.55 6.13	54.38–81.85 40.99–57.68	15.78	8.91	0.001

^a^ Rectal volume in cm^3^; ^b^ Dose in Gy; ^c^ RVXGy = Percentage of rectal volume received x dose; ^d^ Δ difference between the non-ERB plans and the ERB plans (mean value non-ERB plans - mean value ERB plans).

**Table 2:** DVH analysis for the anterior rectum comparing between the non-ERB plans and ERB plans.

	Study Group	Mean	SD	Range	Diff (Δ) ^c^		*p* Value
					Mean	SD	
Ant-D_max_^a^	non-ERB ERB	73.62 72.98	0.77 0.545	72.6–74.8 72.6–74.4	0.64	0.85	0.103
Ant-D1 ^a^	non-ERB ERB	72.77 72.56	0.72 0.23	71.31–73.73 72.30–73.01	0.21	0.69	0.326
Ant-RV72Gy ^b^	non-ERB ERB	5.99 7.13	3.17 2.1	0.66–12.32 3.78–10.29	−1.14	3.71	0.290
Ant-RV70Gy ^b^	non-ERB ERB	14.44 15.39	5.36 3.98	4.98–21.80 8.28–20.98	−0.95	6.49	0.705
Ant-RV65Gy ^b^	non-ERB ERB	22.84 24.05	5.75 5.7	11.39–31.05 14.79–31.02	−1.22	8.41	0.940
Ant-RV60Gy ^b^	non-ERB ERB	31.74 30.81	7.51 6.61	18.49–41.66 21.55–39.18	0.93	9.11	0.545
Ant-RV55Gy ^b^	non-ERB ERB	38.66 37.47	8.32 7.16	25.13–48.53 28.65–48.53	1.19	10.52	0.734
Ant-RV50Gy ^b^	non-ERB ERB	47.34 43.46	7.41 7.29	31.36–56.39 34.42–54.28	3.88	11.14	0.226
Ant-RV40Gy ^b^	non-ERB ERB	59.11 52.51	7.64 8.60	42.45–68.87 42.48–65.22	6.6	11.88	0.174
Ant-RV30Gy ^b^	non-ERB ERB	69.25 61.60	7.9 9.05	52.49–78.39 50.20–76.17	7.65	12.21	0.059
Ant-RV20Gy ^b^	non-ERB ERB	78.84 70.53	9.65 9.03	61.83–89.52 58.28–84.13	8.31	12.81	0.059
Ant-RV10Gy ^b^	non-ERB ERB	85.2 81.03	9.44 7.9	71.7−96.11 68.21−91.01	4.17	11.75	0.174

^a^ Dose in Gy; ^b^ RVXGy =Percentage of ant-rectal volume received X dose; ^c^ Δ difference between the non-ERB plans and the ERB plans (mean value non-ERB plans - mean value ERB plans).

**Table 3:** DVH analysis for the posterior rectum comparing between the non-ERB plans and ERB plans.

	Study Group	Mean	SD	Range	Diff (Δ) ^c^		*p* Value
					Mean	SD	
D_max_^a^	non-ERB ERB	60.24 56.41	9.16 10.54	47.60−72.60 42.80−70.60	3.83	12.41	0.406
D1 ^a^	non-ERB ERB	37.36 72.56	11.82 0.23	23.66−57.94 72.3−73.01	11.11	13.93	0.059
Post-RV72Gy ^b^	non-ERB ERB	0.11 0	0.35 0	0−1.14 0	0.11	0.36	0.317
Post-RV70Gy ^b^	non-ERB ERB	0.51 0	1.6 0	0−5.06 0−0.02	0.51	1.6	0.503
Post-RV65Gy ^b^	non-ERB ERB	1.08 0.03	3.31 0.07	0−10.49 0–0.22	1.06	3.29	0.829
Post-RV60Gy ^b^	non-ERB ERB	1.67 0.11	4.5 0.23	0–14.4 0–0.71	1.56	4.44	0.518
Post-RV55Gy ^b^	non-ERB ERB	2.38 0.24	5.47 0.46	0–17.67 0–1.45	2.14	5.39	0.435
Post-RV50Gy ^b^	non-ERB ERB	3.01 0.451	6.39 0.70	0–20.57 0–2.25	2.55	6.25	0.286
Post-RV40Gy ^b^	non-ERB ERB	4.98 1.148	8.05 1.309	0.2–27.09 0.06–4.03	3.83	7.79	0.069
Post-RV30Gy ^b^	non-ERB ERB	11.59 2.7	10.90 2.26	0.88–36.46 0.33–6.68	8.86	9.92	0.019
Post-RV20Gy ^b^	non-ERB ERB	21.92 6.165	14.84 3.711	3.8–349.00 1.59–10.87	15.76	12.94	0.003
Post-RV10Gy ^b^	non-ERB ERB	39.54 13.88	17.17 6.695	14.87–71.37 3.97–22.05	25.66	14.21	0.001

^a^ Dose in Gy; ^b^ RVXGy =Percentage of post-rectal volume receiving X dose; ^c^ Δ difference between the non-ERB plans and the ERB plans (mean value non-ERB plans - mean value ERB plans).

**Table 4:** NTCP results for the whole rectum comparing between the non-ERB plans and ERB plans.

NTCP ^a^	Study Group	Mean	SD	Range	Diff (Δ) ^b^		*p* Value
					Mean	SD	
Late rectal bleeding ≥ 2	non-ERB ERB	2.6 3.1	0.97 1.1	2–5 1–5	–0.5	1.18	0.150
Necrosis/stenosis	non-ERB ERB	5.5 5.6	1.78 2.22	1–7 1–8	–0.1	2.02	0.728
Late rectal toxicity ≥ 3	non-ERB ERB	13.1 13.3	1.37 3.02	11–15 7–17	–0.2	3.82	0.593

^a^ NTCP results in %; ^b^ Δ difference between the non-ERB plans and the ERB plans (mean value non-ERB plans - mean value ERB plans).

## Data Availability

All data and materials can be accessed via DAK, in compliance with data protection guidelines.

## References

[1] Michalski J.M., Moughan J., Purdy J., Bosch W., Bruner D.W., Bahary J.P., Lau H., Duclos M., Parliament M., Morton G. (2018). Effect of Standard vs Dose-Escalated Radiation Therapy for Patients With Intermediate-Risk Prostate Cancer: The NRG Oncology RTOG 0126 Randomized Clinical Trial. JAMA Oncol..

[2] Kuban D.A., Tucker S.L., Dong L., Starkschall G., Huang E.H., Cheung M.R., Lee A.K., Pollack A. (2008). Long-term results of the M. D. Anderson randomized dose-escalation trial for prostate cancer. Int. J. Radiat. Oncol. Biol. Phys..

[3] Zietman A.L., Bae K., Slater J.D., Shipley W.U., Efstathiou J.A., Coen J.J., Bush D.A., Lunt M., Spiegel D.Y., Skowronski R. (2010). Randomized Trial Comparing Conventional-Dose With High-Dose Conformal Radiation Therapy in Early-Stage Adenocarcinoma of the Prostate: Long-Term Results From Proton Radiation Oncology Group/American College of Radiology 95-09. J. Clin. Oncol..

[4] Heemsbergen W.D., Al-Mamgani A., Slot A., Dielwart M.F., Lebesque J.V. (2014). Long-term results of the Dutch randomized prostate cancer trial: Impact of dose-escalation on local, biochemical, clinical failure, and survival. Radiother. Oncol..

[5] Beckendorf V., Guerif S., Le Prisé E., Cosset J.-M., Bougnoux A., Chauvet B., Salem N., Chapet O., Bourdain S., Bachaud J.-M. (2011). 70 Gy Versus 80 Gy in Localized Prostate Cancer: 5-Year Results of GETUG 06 Randomized Trial. Int. J. Radiat. Oncol. Biol. Phys..

[6] Dearnaley D.P., Jovic G., Syndikus I., Khoo V., Cowan R.A., Graham J.D., Aird E.G., Bottomley D., Huddart R.A., Jose C.C. (2014). Escalated-dose versus control-dose conformal radiotherapy for prostate cancer: Long-term results from the MRC RT01 randomised controlled trial. Lancet Oncol..

[7] Peeters S.T., Heemsbergen W.D., Koper P.C., Van Putten W.L., Slot A., Dielwart M.F., Bonfrer J.M., Incrocci L., Lebesque J.V. (2006). Dose-Response in Radiotherapy for Localized Prostate Cancer: Results of the Dutch Multicenter Randomized Phase III Trial Comparing 68 Gy of Radiotherapy With 78 Gy. J. Clin. Oncol..

[8] Dearnaley D.P., Sydes M.R., Graham J.D., Aird E.G., Bottomley D., Cowan R.A., Huddart R.A., Jose C.C., Matthews J.H., Millar J. (2007). Escalated-dose versus standard-dose conformal radiotherapy in prostate cancer: First results from the MRC RT01 randomised controlled trial. Lancet Oncol..

[9] Delobel J.-B., Gnep K., Ospina J.D., Beckendorf V., Chira C., Zhu J., Bossi A., Messai T., Acosta O., Castelli J. (2017). Nomogram to predict rectal toxicity following prostate cancer radiotherapy. PLoS ONE.

[10] Vargas C., Martinez A., Kestin L.L., Yan D., Grills I., Brabbins D.S., Lockman D.M., Liang J., Gustafson G.S., Chen P.Y. (2005). Dose-volume analysis of predictors for chronic rectal toxicity after treatment of prostate cancer with adaptive image-guided radiotherapy. Int. J. Radiat. Oncol. Biol. Phys..

[11] Kupelian P.A., Reddy C.A., Carlson T.P., Willoughby T.R. (2002). Dose/volume relationship of late rectal bleeding after external beam radiotherapy for localized prostate cancer: Absolute or relative rectal volume?. Cancer J..

[12] Marzi S., Arcangeli G., Saracino B., Petrongari M.G., Bruzzaniti V., Iaccarino G., Landoni V., Soriani A., Benassi M. (2007). Relationships Between Rectal Wall Dose–Volume Constraints and Radiobiologic Indices of Toxicity for Patients With Prostate Cancer. Int. J. Radiat. Oncol..

[13] Ishikawa H., Tsuji H., Kamada T., Hirasawa N., Yanagi T., Mizoe J.-E., Akakura K., Suzuki H., Shimazaki J., Tsujii H. (2006). Risk factors of late rectal bleeding after carbon ion therapy for prostate cancer. Int. J. Radiat. Oncol. Biol. Phys..

[14] Wachter S., Gerstner N., Goldner G., Pötzi R., Wambersie A., Pötter R. (2001). Rectal sequelae after conformal radiotherapy of prostate cancer: Dose-volume histograms as predictive factors. Radiother. Oncol..

[15] Afkhami Ardekani M., Ghaffari H., Navaser M., Zoljalali Moghaddam S.H., Refahi S. (2021). Effectiveness of rectal displacement devices in managing prostate motion: A systematic review. Strahlenther. Onkol..

[16] van Lin E.N., Hoffmann A.L., van Kollenburg P., Leer J.W., Visser A.G. (2005). Rectal wall sparing effect of three different endorectal balloons in 3D conformal and IMRT prostate radiotherapy. Int. J. Radiat. Oncol. Biol. Phys..

[17] Wong A.T., Schreiber D., Agarwal M., Polubarov A., Schwartz D. (2016). Impact of the use of an endorectal balloon on rectal dosimetry during stereotactic body radiation therapy for localized prostate cancer. Pract. Radiat. Oncol..

[18] Teh B.S., Lewis G.D., Mai W., Pino R., Ishiyama H., Butler E.B. (2018). Long-term outcome of a moderately hypofractionated, intensity-modulated radiotherapy approach using an endorectal balloon for patients with localized prostate cancer. Cancer Commun..

[19] Wortel R.C., Oomen-de Hoop E., Heemsbergen W.D., Pos F.J., Incrocci L. (2018). Moderate Hypofractionation in Intermediate- and High-Risk, Localized Prostate Cancer: Health-Related Quality of Life From the Randomized, Phase 3 HYPRO Trial. Int. J. Radiat. Oncol. Biol. Phys..

[20] Deville C., Both S., Bui V., Hwang W.-T., Tan K.-S., Schaer M., Tochner Z., Vapiwala N. (2012). Acute gastrointestinal and genitourinary toxicity of image-guided intensity modulated radiation therapy for prostate cancer using a daily water-filled endorectal balloon. Radiat. Oncol..

[21] Vlachaki M.T., Teslow T.N., Ahmad S. (2007). Impact of Endorectal Balloon in the Dosimetry of Prostate and Surrounding Tissues in Prostate Cancer Patients Treated with IMRT. Med. Dosim..

[22] Smeenk R.J., van Lin E.N., van Kollenburg P., Kunze-Busch M., Kaanders J.H. (2009). Anal wall sparing effect of an endorectal balloon in 3D conformal and intensity-modulated prostate radiotherapy. Radiother. Oncol..

[23] Patel R.R., Orton N., Tomé W.A., Chappell R., Ritter M.A. (2003). Rectal dose sparing with a balloon catheter and ultrasound localization in conformal radiation therapy for prostate cancer. Radiother. Oncol..

[24] Vargas C., Mahajan C., Fryer A., Indelicato D., Henderson R.H., McKenzie C., Horne D., Chellini A., Lawlor P., Li Z. (2007). Rectal Dose–Volume Differences Using Proton Radiotherapy and a Rectal Balloon or Water Alone for the Treatment of Prostate Cancer. Int. J. Radiat. Oncol. Biol. Phys..

[25] Wootton L.S., Kudchadker R.J., Beddar A.S., Lee A.K. (2012). Effectiveness of a novel gas-release endorectal balloon in the removal of rectal gas for prostate proton radiation therapy. J. Appl. Clin. Med. Phys..

[26] Hedrick S.G., Fagundes M., Robison B., Blakey M., Renegar J., Artz M., Schreuder N. (2017). A comparison between hydrogel spacer and endorectal balloon: An analysis of intrafraction prostate motion during proton therapy. J. Appl. Clin. Med. Phys..

[27] Agren Cronqvist A.K., Kallman P., Turesson I., Brahme A. (1995). Volume and heterogeneity dependence of the dose-response relationship for head and neck tumours. Acta Oncol..

[28] Vanneste B.G.L., van Wijk Y., Lutgens L.C., Van Limbergen E.J., van Lin E.N., van de Beek K., Lambin P., Hoffmann A.L. (2018). Dynamics of rectal balloon implant shrinkage in prostate VMAT: Influence on anorectal dose and late rectal complication risk. Strahlenther. Onkol..

[29] Qamhiyeh S., Geismar D., Pöttgen C., Stuschke M., Farr J. (2012). The effects of motion on the dose distribution of proton radiotherapy for prostate cancer. J. Appl. Clin. Med. Phys..

[30] Rancati T., Fiorino C., Gagliardi G., Cattaneo G.M., Sanguineti G., Borca V.C., Cozzarini C., Fellin G., Foppiano F., Girelli G. (2004). Fitting late rectal bleeding data using different NTCP models: Results from an Italian multi-centric study (AIROPROS0101). Radiother. Oncol..

[31] Dale E., Hellebust T.P., Skjønsberg A., Høgberg T., Olsen D.R. (2000). Modeling normal tissue complication probability from repetitive computed tomography scans during fractionated high-dose-rate brachytherapy and external beam radiotherapy of the uterine cervix. Int. J. Radiat. Oncol. Biol. Phys..

[32] Elsayed H., Bolling T., Moustakis C., Müller S.-B., Schüller P., Ernst I., Willich N., Könemann S. (2007). Organ Movements and Dose Exposures in Teletherapy of Prostate Cancer using a Rectal Balloon. Strahlenther. Onkol..

[33] Hille A., Schmidberger H., Töws N., Weiss E., Vorwerk H., Hess C.F. (2005). The Impact of Varying Volumes in Rectal Balloons on Rectal Dose Sparing in Conformal Radiation Therapy of Prostate Cancer. A prospective three-dimensional analysis. Strahlenther. Onkol..

[34] Wachter S., Gerstner N., Dorner D., Goldner G., Colotto A., Wambersie A., Pötter R. (2002). The influence of a rectal balloon tube as internal immobilization device on variations of volumes and dose-volume histograms during treatment course of conformal radiotherapy for prostate cancer. Int. J. Radiat. Oncol. Biol. Phys..

[35] Storey M.R., Pollack A., Zagars G., Smith L., Antolak J., Rosen I. (2000). Complications from radiotherapy dose escalation in prostate cancer: Preliminary results of a randomized trial. Int. J. Radiat. Oncol. Biol. Phys..

[36] Huang E.H., Pollack A., Levy L., Starkschall G., Dong L., Rosen I., Kuban D.A. (2002). Late rectal toxicity: Dose-volume effects of conformal radiotherapy for prostate cancer. Int. J. Radiat. Oncol. Biol. Phys..

[37] Zapatero A., García-Vicente F., Modolell I., Alcántara P., Floriano A., Cruz-Conde A., Torres J.J., Pérez-Torrubia A. (2004). Impact of mean rectal dose on late rectal bleeding after conformal radiotherapy for prostate cancer: Dose–volume effect. Int. J. Radiat. Oncol. Biol. Phys..

[38] Tucker S.L., Dong L., Cheung R., Johnson J., Mohan R., Huang E.H., Liu H.H., Thames H.D., Kuban D. (2004). Comparison of rectal dose–wall histogram versus dose–volume histogram for modeling the incidence of late rectal bleeding after radiotherapy. Int. J. Radiat. Oncol. Biol. Phys..

[39] Jackson A., Skwarchuk M.W., Zelefsky M.J., Cowen D.M., Venkatraman E.S., Levegrun S., Burman C.M., Kutcher G.J., Fuks Z., Liebel S.A. (2001). Late rectal bleeding after conformal radiotherapy of prostate cancer. II. Volume effects and dose-volume histograms. Int. J. Radiat. Oncol. Biol. Phys..

